# Supplementary material for: Visual attention modulates the integration of goal-relevant evidence and not value
Source: eLife. 2020 Nov 17;9:e60705. doi: 10.7554/eLife.60705 (PMC7723413; doi:10.7554/eLife.60705)
Supplement: Transparent reporting form [file elife-60705-transrepform.docx]

***eLife’s* transparent reporting form**

We encourage authors to provide detailed information *within their submission* to facilitate the interpretation and replication of experiments. Authors can upload supporting documentation to indicate the use of appropriate reporting guidelines for health-related research (see [EQUATOR Network](http://www.equator-network.org/%20)), life science research (see the [BioSharing Information Resource](https://biosharing.org/)), or the [ARRIVE guidelines](http://www.plosbiology.org/article/info:doi/10.1371/journal.pbio.1000412) for reporting work involving animal research. Where applicable, authors should refer to any relevant reporting standards documents in this form.

If you have any questions, please consult our Journal Policies and/or contact us: [editorial@elifesciences.org](mailto:editorial@elifesciences.org).

**Sample-size estimation**

- You should state whether an appropriate sample size was computed when the study was being designed
- You should state the statistical method of sample size computation and any required assumptions
- If no explicit power analysis was used, you should describe how you decided what sample (replicate) size (number) to use

Please outline where this information can be found within the submission (e.g., sections or figure legends), or explain why this information doesn’t apply to your submission:

Our initial objective was to replicate the attentional effect reported in the first experiment of Folke et al. (2016): the influence of difference in dwell time (∆DT) over choice. We calculated the required sample size to generate a large effect size (Cohen’s f2 >0.35) of a single parameter (∆DT) in a hierarchical multiple regression. Following the most parsimonious choice model in Folke et al. (2016) 5 additional predictors of choice (∆Value, confidence, ΣValue, ∆ValuexConfidence, ∆ValuexΣValue) were included in the estimation. Considering a desired power level of 0.8 and p<0.05 we found the minimum required sample size was of 30 participants. We also estimated the required sample to replicate the effect that ΣValue has over confidence, reported also in a hierarchical multiple regression in Folke et al. (2016). Anticipating a large effect size and with 3 additional predictors (|DValue|, RT, ΣValue), power level = 0.8 and p<0.05 we found 28 participants would be required.

**Replicates**

- You should report how often each experiment was performed
- You should include a definition of biological versus technical replication
- The data obtained should be provided and sufficient information should be provided to indicate the number of independent biological and/or technical replicates
- If you encountered any outliers, you should describe how these were handled
- Criteria for exclusion/inclusion of data should be clearly stated
- High-throughput sequence data should be uploaded before submission, with a private link for reviewers provided (these are available from both GEO and ArrayExpress)

Please outline where this information can be found within the submission (e.g., sections or figure legends), or explain why this information doesn’t apply to your submission:

Details about experimental setup and participants are presented in Methods section (4.1 and 4.3). Criteria for participant exclusion is detailed in section 4.2. Other aspects of replication described above do not apply to our study since it is a human behavioural study.

**Statistical reporting**

- Statistical analysis methods should be described and justified
- Raw data should be presented in figures whenever informative to do so (typically when N per group is less than 10)
- For each experiment, you should identify the statistical tests used, exact values of N, definitions of center, methods of multiple test correction, and dispersion and precision measures (e.g., mean, median, SD, SEM, confidence intervals; and, for the major substantive results, a measure of effect size (e.g., Pearson's r, Cohen's d)
- Report exact p-values wherever possible alongside the summary statistics and 95% confidence intervals. These should be reported for all key questions and not only when the p-value is less than 0.05.

Please outline where this information can be found within the submission (e.g., sections or figure legends), or explain why this information doesn’t apply to your submission:

Sample sizes used in our analysis in value and perceptual experiments are reported in the Methods section. Figures in the manuscript present individual participants and group averages (e.g. figure 2 and 4). In the main text and figure legends we indicate statistical tests and precision measures resulting from our analysis. Additional tables with details of statistical results are available in the supplemental information (e.g. tables S2 and S3 for the statistical results of choice regression analysis).

(For large datasets, or papers with a very large number of statistical tests, you may upload a single table file with tests, Ns, etc., with reference to sections in the manuscript.)

**Group allocation**

- Indicate how samples were allocated into experimental groups (in the case of clinical studies, please specify allocation to treatment method); if randomization was used, please also state if restricted randomization was applied
- Indicate if masking was used during group allocation, data collection and/or data analysis

Please outline where this information can be found within the submission (e.g., sections or figure legends), or explain why this information doesn’t apply to your submission:

Independent participants samples were recruited for our value and perceptual experiments. Our analysis was based on a within participant comparison for two framing conditions, therefore group allocation was not a consideration in the design.

**Additional data files (“source data”)**

- We encourage you to upload relevant additional data files, such as numerical data that are represented as a graph in a figure, or as a summary table
- Where provided, these should be in the most useful format, and they can be uploaded as “Source data” files linked to a main figure or table
- Include model definition files including the full list of parameters used
- Include code used for data analysis (e.g., R, MatLab)
- Avoid stating that data files are “available upon request”

Please indicate the figures or tables for which source data files have been provided:

Experimental dataset and analysis code for value and perceptual experiments, including computational models, are available in the Brain Decision Modelling (BDM) lab GitHub (<https://github.com/BDMLab>). Experiment datasets are organized in csv files. Python code and Jupyter notebooks contain analysis and models.
